# Cholesterol Granuloma of the Lower Lip: Report of a Rare Case

**DOI:** 10.30476/dentjods.2024.100683.2242

**Published:** 2024-09-01

**Authors:** Sorena Fardisi, Sara Amanpour

**Affiliations:** 1 Dept. of Oral and Maxillofacial Surgery, School of Dentistry, Kerman University of Medical Sciences, Kerman, Iran; 2 Dept. of Oral and Maxillofacial Pathology, Faculty of Dentistry, Kerman University of Medical Sciences, Kerman, Iran

**Keywords:** Granuloma, Foreign body, Lower lip, Case report

## Abstract

Cholesterol granuloma is the result of foreign body type response to the deposition of cholesterol crystals in the tissues. It is usually associated with chronic middle ear diseases and the middle ear and mastoid antrum are the most common location for this lesion. Histopathological findings are accumulation of cholesterol clefts, ghost cells, chronic inflammatory cells, and giant cells in a fibrous granulation tissue. Cases of cholesterol granuloma have been recently reported in the jaws but still they are few in the literature of dentistry. This article presents a unique case of cholesterol granuloma occurring in the lower lip secondary to a history of trauma.

## Introduction

Cholesterol granuloma is a pathological lesion commonly associated with chronic middle ear diseases. It results from the aggregation of cholesterol crystals and is primarily located in the mastoid antrum, air cells of the temporal bone, and occasionally in the skull, paranasal sinuses [ 1 - 2 ], and in association with certain odontogenic cysts [ 3 - 5 ]. This condition occurs more frequently in middle-aged men than in women, with a male-to-female ratio of 3:1 [ 1 ]. Trauma and bleeding are considered the main etiological factors. Inadequate drainage leads to the precipitation of cholesterol in crystalline form and connective tissue degeneration may also play a role [ 6 ].

Although the presence of cholesterol granuloma in paranasal sinuses [ 2 ], in nasopalatine area [ 1 ], and in association with jaw cysts [ 3 - 5 ] is well-documented in the literature, this case represents the first case of cholesterol granulomas occurring in the labial mucosa secondary to a history of trauma.

## Case Presentation

A 56-year-old man was referred to the Maxillofacial Surgery Clinic of Kerman University of Medical Sciences in 2023 with a painless swelling in the lower lip mucosa that has been present for one month. The patient had a history of falling before the onset of the lip swelling, which had not significantly increased in size since its appearance. Clinical examination revealed a dome-shaped swelling measuring 8 × 8 mm, covered by intact mucosa with a color similar to adjacent mucosa and a soft consistency in the lower lip mucosa. There were no enlarged cervical lymph nodes. Owing to the location of the lesion, clinical presentation, and patient history, a differential diagnosis of a mucocele was considered. 

The lesion was surgically excised and the specimen was sent to the pathological laboratory.

**Figure 1:**
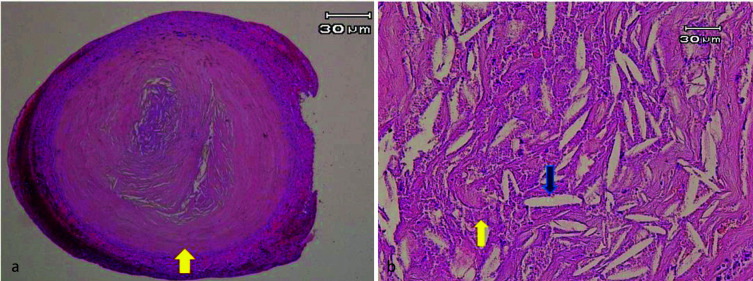
**a:** A well-defined lesion composed of granulation tissue, collagen fibers (arrow) and deposition of cholesterol clefts in hematoxylin and eosin staining (H&E/ 40×), **b:** Multiple cholesterol clefts (blue arrow) intermixed with ghost cells (yellow arrow) in hematoxylin and eosin staining (H&E/ 400×)Multiple cholesterol clefts (blue arrow) intermixed with ghost cells (yellow arrow) in hematoxylin and eosin staining (H&E/ 400×)

Histological examination was done by using hematoxylin and eosin staining (H&E/ X 40, 400) and revealed a small soft tissue mass composed of numerous cholesterol clefts, chronic inflammatory cells, ghost cells, and hemosiderin-laden macrophages in the center being embedded in fibrous granulation tissue surrounded by collagen fibers at the periphery of the lesion (Figure 1a-b). Based on this feature, a final diagnosis of cholesterol granuloma was made.

## Discussion

Cholesterol granuloma is a rare entity that occurs in response to the deposition of cholesterol crystals [ 6 ].

The known etiological factors include hemorrhage and impaired drainage [ 2 ]. In an inflammatory context, macrophages scavenge low-density lipoproteins and convert them into lipid-laden foamy cells that rupture and release concentrated lipids into the extracellular space. This leads to the crystallization of cholesterol [ 7 ]. The deposited cholesterol crystals act as a foreign body and attract macrophages and giant cells into the affected area. Macrophages transform hydrophobic cholesterol crystals into a soluble form by integrating them into a lipoprotein carrier. However, when the crystals grow in size, they become resistant to being internalized by macrophages. This resistance leads to the formation of multinucleated giant cells through a process called circumfusion. These giant cells can persist for extended periods, but they struggle to break down cholesterol, leading to the release of inflammatory and bone-resorbing substances. This, in turn, exacerbates bone loss and the expansion of the lesion [ 5 ].

In the jaw region, cholesterol granulomas mainly occur in paranasal sinuses and in association with some cysts [ 2 - 5 ]. In fact, cholesterol crystals are more common in inflammatory cysts, particularly in radicular cysts and show a relatively low incidence of developmental cysts [ 8 ]. According to two comprehensive reviews of reported cases in the paranasal sinuses, the mean age of affected patients is approximately 43.8 [ 9 ] and 45.4 years [ 2 ], with a higher prevalence in men [ 2 ]. The frontal sinus is the most commonly affected location, followed by the maxillary sinus, ethmoid sinus, and sphenoid sinus [ 2 ]. 

Herein, we have reported a unique occurrence of cholesterol granuloma in oral mucosa. Clinical features and a history of trauma initially led to a differential diagnosis of a mucocele, which was logical. However, microscopic examination revealed the rare entity of cholesterol granuloma, which can develop due to trauma and subsequent hemorrhage in the affected area. This case was reported with the informed consent of the patient.

## Conclusion

In conclusion, cholesterol granuloma represents a foreign body reaction to the presence of cholesterol crystals and may manifest in various anatomical sites in the body. We reported the first documented case of cholesterol granuloma occurring in soft tissue in the oral cavity.

This case underscores that cholesterol granulomas can mimic other soft tissue pathologies on their clinical presentation. Though rare, it should be considered in the differential diagnosis of soft tissue lesions.
